# Human Versus Machine in Survival Prediction for Metastatic Spinal Cord Compression: A Retrospective Cohort Study

**DOI:** 10.7759/cureus.108858

**Published:** 2026-05-14

**Authors:** Vaishnavi Sharma, Rawan Masarwa, Jonathan Dimitry, Khalid Salem, Nasir A Quraishi, Elie Najjar

**Affiliations:** 1 Centre for Spinal Studies and Surgery, Queens Medical Centre, Nottingham University Hospitals NHS Trust, Nottingham, GBR

**Keywords:** ai and machine learning, metastatic spinal cord compression, prognostic modelling, survival analysis, treatment decision-making

## Abstract

Background: Accurate survival prediction in metastatic spinal cord compression (MSCC) is critical for guiding treatment decisions, yet remains challenging, particularly for intermediate survival durations. We compared the accuracy of oncologist judgment, surgeon-calculated Tokuhashi scores, and ChatGPT-assisted predictions in estimating survival outcomes in MSCC patients.

Methods: This retrospective study included 100 patients (n = 100) referred to the Centre for Spinal Studies and Surgery, Queen’s Medical Centre, a tertiary spinal oncology center in Nottingham, United Kingdom, with radiologically confirmed MSCC. Anonymized clinical data were used to calculate surgeon Tokuhashi scores, document oncologist-estimated life expectancy, and generate ChatGPT-assisted survival predictions based on both literature review and Tokuhashi calculation. Predictions were compared against actual survival outcomes (<6 months, six to 12 months, >12 months). Machine learning analyses identified key predictors of survival.

Results: Overall prediction accuracy was 53% for ChatGPT Tokuhashi-based predictions, 49% for surgeon Tokuhashi scores, 47% for oncologist judgment, and 36% for ChatGPT literature-based estimates. Recall for short survival (<6 months) was the highest with the surgeon (70%) and ChatGPT Tokuhashi (68%) methods, whereas intermediate survival (six to 12 months) remained difficult to predict across all modalities. For long-term survival (>12 months), oncologists performed better (74% recall). Functional status (Karnofsky score) and patient age emerged as the strongest survival predictors across logistic regression, random forest, decision tree, and XGBoost models, surpassing primary tumor type and metastasis burden.

Conclusions: Structured prognostic tools and AI-assisted scoring can complement clinical judgment in predicting short-term survival in MSCC. However, intermediate-term survival prediction remains a critical unmet need. Future prognostic strategies should prioritize dynamic functional metrics over static tumor classifications to improve personalized decision-making.

## Introduction

Accurate prediction of life expectancy in patients with metastatic spinal cord compression (MSCC) is essential for guiding treatment selection, balancing surgical risk, and setting realistic patient expectations [1]. In this vulnerable patient population, survival projections directly influence treatment options [2], with thresholds such as six and 12 months often serving as critical decision points for surgical intervention, radiotherapy, or palliative care strategies [3]. However, survival estimation remains inherently challenging [4], influenced by a complex interplay of oncological, functional, and systemic factors [5,6].

Traditional prognostic tools, such as the revised Tokuhashi score, have historically provided structured frameworks to guide decision-making [6]. While such scoring systems facilitate systematic evaluation, they were developed prior to the widespread adoption of targeted therapies and immunotherapy and may not fully account for contemporary oncological advances [6]. Notably, recent studies have suggested only modest predictive accuracy for Tokuhashi-based estimations, with overall survival prediction rates approximating 60-70% [6]. Although clinical judgment by oncologists incorporates nuanced assessments beyond formal scoring, recent studies have demonstrated persistent limitations in predictive accuracy, particularly in forecasting intermediate survival periods [7].

Recent efforts to integrate artificial intelligence (AI) and machine learning (ML) into oncology prognostication have shown promise in areas such as tumor detection and other image-based classification tasks [8-10]. However, while most AI research in spinal oncology has centered around imaging classification tasks, structured prediction models based on clinical data remain underexplored [11-12]. Moreover, external validation of AI models has revealed substantial variability in performance, underscoring concerns regarding generalizability across diverse patient populations [9,13,14].

Importantly, few studies have critically addressed the specific difficulty of predicting survival in the intermediate range (six to 12 months), a group in which clinical decisions are often most uncertain, and where misclassification can have profound consequences [2,3]. Existing literature has largely emphasized overall accuracy metrics, without dissecting the variable performance across survival strata [6,7]. Other evaluations of prognostic modelling have demonstrated the limitations of utilising only global discrimination metrics without analysis of subgroups [14].

Using a real-world cohort of anonymized clinical cases encompassing oncological, imaging, and functional parameters, the primary objective of this study was to compare the accuracy of survival prediction in MSCC across four prognostic approaches: oncologist clinical judgment, surgeon-calculated Tokuhashi scores, ChatGPT-assisted literature-based predictions, and ChatGPT-assisted Tokuhashi-based predictions. We specifically assessed performance within clinically relevant survival categories (<6 months, six to 12 months, and >12 months). Secondary exploratory objectives were to examine areas of relative agreement and difficulty across these prognostic approaches and to explore potential predictors of survival using logistic regression and machine learning analyses. Through this approach, we aimed to provide a clinically relevant assessment of current and emerging prognostic strategies in metastatic spinal disease.

## Materials and methods

Study design and patient selection

We conducted a retrospective cohort study at the Centre for Spinal Studies and Surgery, Queen’s Medical Centre, Nottingham, United Kingdom. Consecutive patients with MSCC referred to our tertiary spinal oncology service between January 2020 and December 2022 were included. A total of 100 patients (n = 100) were analyzed. Referral data routinely included the oncology team's initial management plan and predicted life expectancy at the time of referral.

As all data were fully anonymized and retrospective in nature, institutional policy determined that formal ethical approval was not required.

Data collection

**Figure 1:**
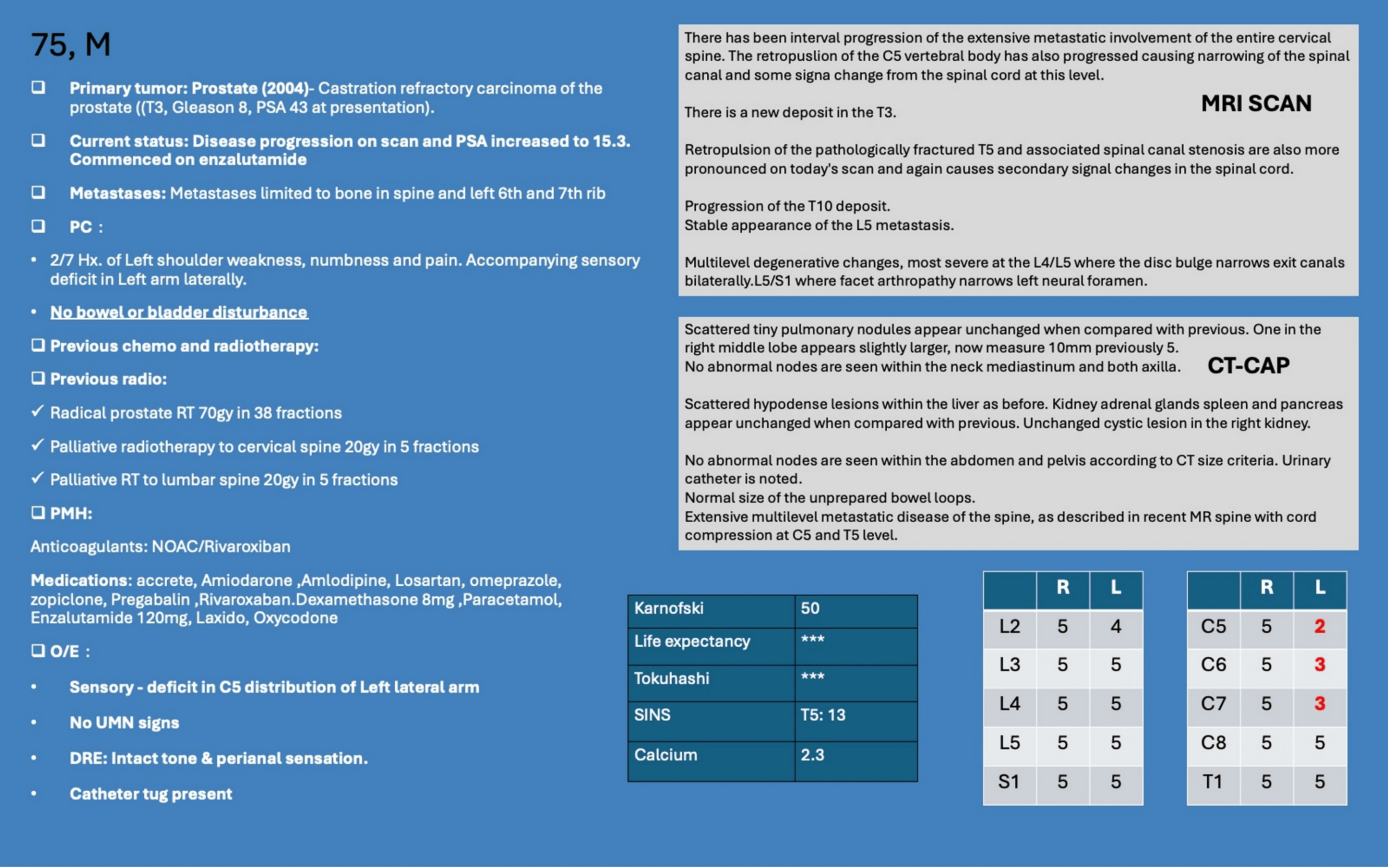
Example of an anonymized clinical slide used for prognostic assessment The figure was created by the authors with Microsoft PowerPoint (Microsoft Corp., USA).

Clinical data were summarized into standardized PowerPoint slides (Microsoft Corp., USA) for each patient. Each slide included patient demographics particularly age and gender, presenting complaint, past medical history, primary tumor histology and systemic metastases, imaging findings including CT chest/abdomen/pelvis and MRI whole spine, biopsy results whenever they were available, Karnofsky Performance Status, serum calcium levels, Spinal Instability Neoplastic Score (SINS), clinical neurological examination findings, current medications, and previous chemotherapy and radiotherapy treatments. All slides were anonymized to exclude any identifiable patient information. Figure 1 illustrates an example of the anonymized clinical slide used for prognostic assessment.

Prognostic estimation

Pertaining to prognostication, for each patient, three prognostic assessments were documented. The first assessment was the oncologist-predicted life expectancy as recorded at the time of referral. The second assessment was the Tokuhashi scores calculated independently by spinal fellows based on the clinical data provided, using the revised Tokuhashi scoring criteria. The third prognostication assessment was ChatGPT-based estimations, obtained in two stages. The first stage was literature-based prediction: ChatGPT-4.5 (OpenAI, San Francisco, CA, USA; accessed March 2025) was first prompted to predict life expectancy after reviewing the anonymized slide and performing a literature-based interpretation. The second stage was Tokuhashi-based prediction in which ChatGPT was then prompted to independently calculate a Tokuhashi score from the clinical information and predict survival based on published Tokuhashi thresholds. Predictions were generated across at least five separate chat sessions.

To ensure reproducibility and minimize variability in AI responses, ChatGPT was queried using a standardized prompt format following each anonymized clinical slide, requesting life expectancy estimation based on structured clinical analysis. To further enhance consistency and accurate interpretation, one example slide was initially used to calibrate ChatGPT’s recognition of abbreviations, clinical tables, and formatting conventions before formal predictions were conducted. Importantly, neither the oncologist-predicted life expectancy nor the surgeon-calculated Tokuhashi scores were included in the slides reviewed by ChatGPT. Cases with missing variables were excluded; therefore, ChatGPT predictions were based only on slides containing complete study variables, and no imputation of missing data was performed.

The prompts used are available in the Appendix. 

Survival data

Actual survival outcomes were collected by reviewing hospital records, oncology clinic notes, and death registry data. Survival was categorized into three groups: <6 months, six to 12 months, and >12 months.

Statistical analysis

Prediction performance was evaluated by comparing estimated survival categories to actual survival outcomes. Performance metrics included overall accuracy, recall (sensitivity), precision (positive predictive value), contingency table-based analysis, and Cohen’s kappa coefficients for inter-method agreement.

In addition, logistic regression, decision tree, random forest, and XGBoost machine learning models were developed to identify the most influential predictors of survival based on the clinical variables. Feature importance analysis was performed to rank predictors within each model.

The chi-square test was performed using the full contingency table of the predicted versus actual survival categories across all methods to assess differences in prediction distributions. A p-value < 0.05 was considered statistically significant. All statistical analyses were performed using Python (v3.10), scikit-learn (v1.3), and SciPy.

## Results

Patient cohort

**Figure 2:**
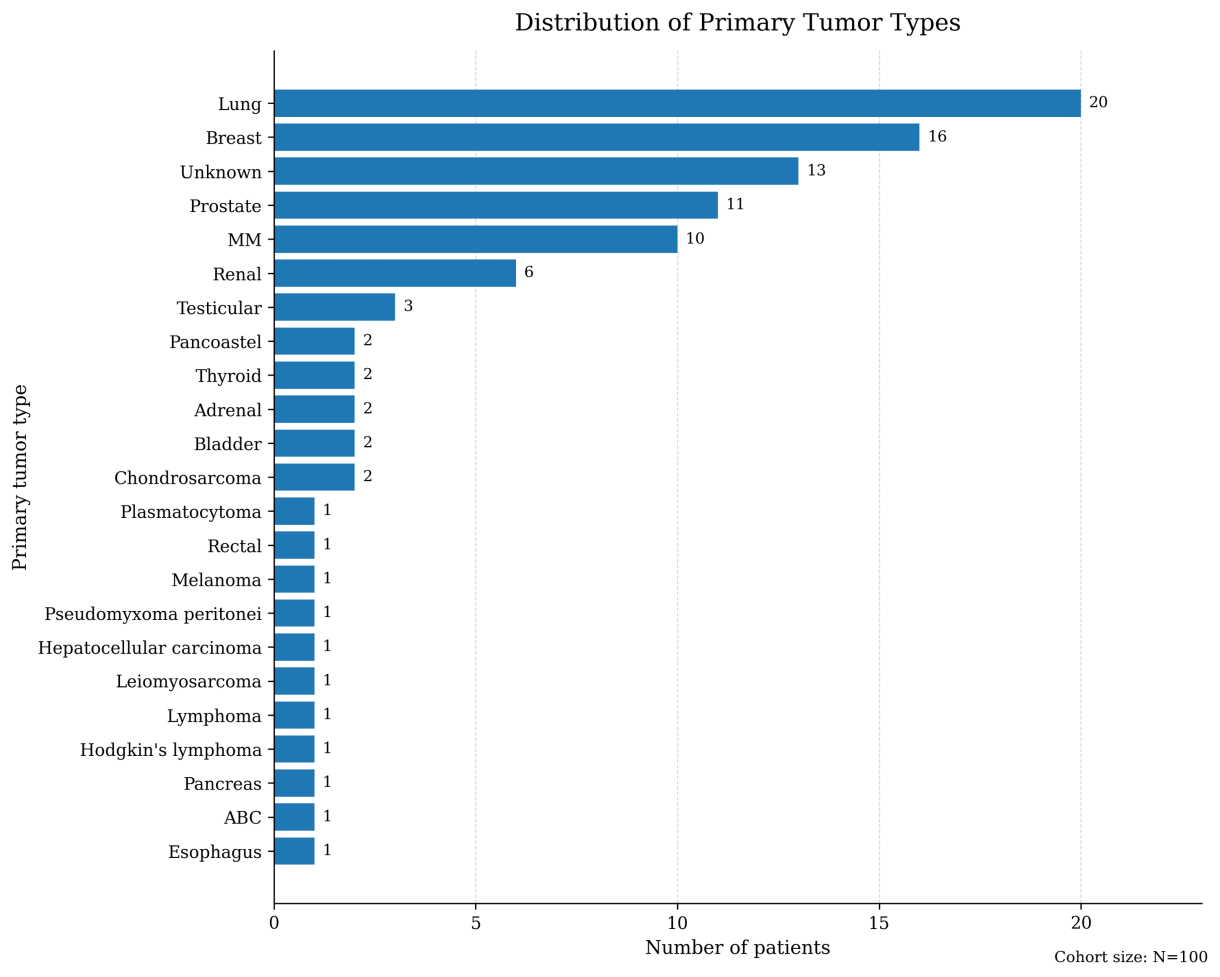
Distribution of primary tumor types MM: multiple myeloma, ABC: aneurysmal bone cyst This figure was created by the authors using Python (Python Software Foundation, USA).

A total of 100 patients (n = 100) with MSCC were included in the analysis. The cohort comprised both male and female patients across a wide age range, with prostate, breast, and lung cancers representing the most common primary tumors. All patients had radiologically confirmed MSCC at presentation. The baseline patient characteristics are summarized in Table 1 and Figure 2.

**Table 1:** Baseline patient characteristics (n = 100). Continuous variables are presented as mean ± SD; categorical variables as n (%). ASIA: American Spinal Injury Association

Variable	Value
Age (years)	67.4 ± 11.1
Karnofsky Performance Status	73.1 ± 18.5
Number of spine levels involved	4.8 ± 3.3
Male	52 (52%)
Female	48 (48%)
Primary tumor – prostate	23 (23%)
Primary tumor – breast	22 (22%)
Primary tumor – lung	15 (15%)
Metastatic disease present	76 (76%)
No systemic metastases	24 (24%)
ASIA E	72 (72%)
ASIA D	25 (25%)
ASIA C	2 (2%)
ASIA A	1 (1%)
Survival <6 months	56 (56%)
Survival six to 12 months	13 (13%)
Survival >12 months	31 (31%)

Overall, most patients demonstrated preserved neurological function and a high burden of systemic metastatic disease. Regarding survival outcomes, 56 (56%) patients survived <6 months, 13 (13%) survived six to 12 months, and 31 (31%) survived >12 months.

Prediction performance

Overall prediction performance across methods is summarized in Table 2. ChatGPT Tokuhashi-based prediction showed the highest overall accuracy, followed by surgeon-calculated Tokuhashi, oncologist clinical judgment, and ChatGPT literature-based prediction. Differences in overall prediction accuracy between methods were assessed using the chi-square test (χ² = 6.38, p = 0.095), with statistical significance defined as p < 0.05. No significant differences were observed. Agreement with actual survival, measured by Cohen’s kappa, was fair for oncologist judgment (κ = 0.26) and ChatGPT Tokuhashi (κ = 0.22), and slight for surgeon Tokuhashi (κ = 0.16) and ChatGPT literature-based estimates (κ = 0.10).

**Table 2:** Overall prediction performance across methods Accuracy is presented as number of correct predictions (n) and percentage (%). Differences between methods were assessed using the chi-square test (χ² = 6.38, p = 0.095).

Method	Accuracy
Oncologist	47 (47%)
Surgeon Tokuhashi	49 (49%)
ChatGPT Tokuhashi	53 (53%)
ChatGPT literature-based	36 (36%)

**Figure 3:**
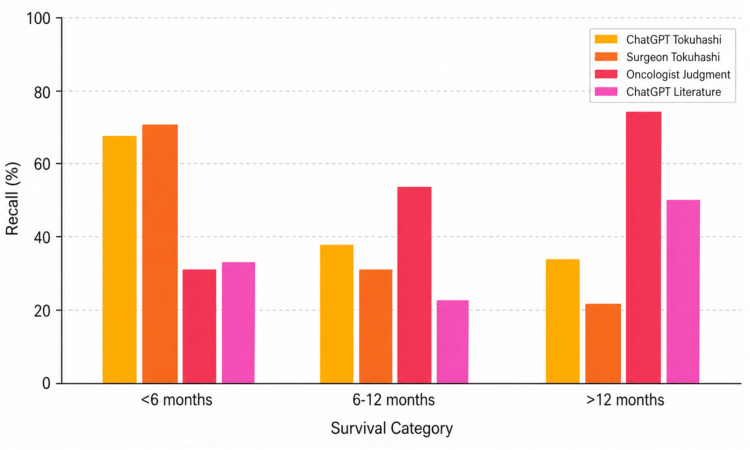
Recall by survival group (<6 months, six to 12 months, >12 months) across methods. Data are presented as percentages (%). Differences in prediction distribution were assessed using the chi-square test (χ² = 119.47, p < 0.001).

When stratified by actual survival outcomes, important differences emerged. The distribution of predicted survival categories across methods and actual survival groups is presented in Table 3. Recall by survival group across methods is illustrated in Figure 3.

**Table 3:** Distribution of predicted survival categories across methods and actual survival groups Values are presented as number of cases (n). Each row represents actual survival and columns represent predicted survival categories. Differences in the distribution of predicted survival categories were assessed using the chi-square test (χ² = 119.47, p < 0.001).

Method	Actual survival	Predicted <6 months (n)	Predicted six to 12 months (n)	Predicted >12 months (n)
Oncologist	<6 months	17	25	13
Oncologist	Six to 12 months	2	7	4
Oncologist	>12 months	2	6	23
Surgeon Tokuhashi	<6 months	39	15	1
Surgeon Tokuhashi	Six to 12 months	8	4	1
Surgeon Tokuhashi	>12 months	12	13	6
ChatGPT Tokuhashi	<6 months	38	10	7
ChatGPT Tokuhashi	6–12 months	6	5	2
ChatGPT Tokuhashi	>12 months	12	9	10
ChatGPT literature-based	<6 months	18	23	14
ChatGPT literature-based	6-12 months	3	3	7
ChatGPT literature-based	>12 months	2	14	15

For short-term survival (<6 months), surgeon-calculated Tokuhashi and ChatGPT Tokuhashi provided the highest recall (70% and 68%, respectively), markedly outperforming oncologist judgment (30%) and ChatGPT literature-based prediction (32%). For intermediate survival (six to 12 months), predictive performance was poor across all methods, with recall ranging from 23% to 54%. No method demonstrated consistent reliability within this subgroup. For long-term survival (>12 months), oncologists performed better (74% recall), whereas Tokuhashi-based predictions, both human and AI-assisted, showed lower sensitivity (19% and 32%, respectively). ChatGPT literature-based predictions demonstrated moderate performance (48% recall). Notably, misclassification was most frequent among patients who survived 6-12 months, confirming this intermediate group as the most difficult to predict reliably, regardless of prognostic approach; however, this should be interpreted cautiously given the small subgroup size. This subgroup represents a critical clinical challenge, as treatment decisions-particularly regarding surgical intervention-are highly sensitive to survival estimates in this timeframe. 

Prediction model coefficients for survival categories are detailed in Table 4. Functional status, as measured by Karnofsky Performance Status, was associated with longer survival, while the presence and extent of metastatic disease showed the strongest association with short-term survival. Age and MSCC demonstrated comparatively smaller effects across survival categories.

**Table 4:** Logistic regression coefficients for predictors of survival in metastatic spinal cord compression. Class 1 refers to patients who survived <6 months, Class 2 are patients that survived six to 12 months and class 3 are patients that survived >12 months. Values represent regression coefficients for each predictor across survival categories. ASIA: American Spinal Injury Association, MSCC: metastatic spinal cord compression

Predictor	Class 1 vs. rest (<6 months)	Class 2 vs. rest (six to 12 months)	Class 3 vs. rest (>12 months)
ASIA	+0.31	−0.73	+0.42
Age	−0.02	−0.03	+0.04
Karnofsky	−0.04	+0.01	+0.02
MSCC	−0.03	−0.01	+0.03
Metastasis (Y/N)	+0.43	+0.05	−0.48
Metastasis count	+0.09	+0.31	−0.40
Spine levels	+0.01	−0.15	+0.13

Exploratory machine learning feature importance 

Machine learning exploratory analyses using decision tree, random forest, and XGBoost models were utilized to capture potential non-linear relationships and interactions between predictors. Across the exploratory machine learning analyses, functional status (Karnofsky score) and age appeared to be the most influential predictors of survival within this cohort, as shown in Table 5 and Table 6. By contrast, traditional oncological factors such as primary tumor histology, number of vertebral metastases, and presence of MSCC showed minimal predictive value.

**Table 5:** Feature importance rankings from random forest and decision tree models predicting survival in metastatic spinal cord compression. Values are presented as percentage (%), representing the relative contribution of each variable to model predictions, normalized to sum to 100%. ASIA: American Spinal Injury Association, MSCC: metastatic spinal cord compression

Feature	Random forest importance (%)	Decision tree importance (%)
Karnofsky	45	42
Age	30	32
Primary tumor	10	11
Metastasis burden	8	9
MSCC presence	4	3
ASIA grade	3	3

**Table 6:** Feature importance rankings from XGBoost model predicting survival in metastatic spinal cord compression. ASIA: American Spinal Injury Association, MSCC: metastatic spinal cord compression

Feature	Importance
Karnofsky	Most important
Age	Strong predictor
Spine levels involved	High impact
Metastasis count	Moderate
ASIA	Lower
Metastasis (Y/N)	Minimal
MSCC	Negligible

## Discussion

Accurate survival prediction in MSCC remains a central challenge in spine oncology. In this study, we critically compared survival estimations made by oncologists, surgeon-calculated Tokuhashi scores, and ChatGPT-assisted predictions using a real-world cohort of MSCC patients. Our findings highlight that while prediction accuracy remains modest across all methods, Tokuhashi-based predictions, both human and AI-assisted, outperformed oncologist judgment for short-term survival (<6 months), while for long-term survival (>12 months), oncologists performed better. Intermediate-term survival (six to 12 months) continues to represent a major predictive gap. Although differences in overall prediction accuracy were observed between methods, these did not reach statistical significance. By contrast, the distribution of predicted survival categories differed significantly across survival groups and methods. This emphasizes substantial variability in predictive performance across methods depending on the survival category. This suggests that while overall prediction accuracy of the various methods may be comparable, clinically significant differences emerge when performance is examined within specific survival groups. 

These results align with recent findings from Cox et al. [7], who demonstrated that oncologists predict short-term survival with reasonable sensitivity but struggle significantly in the intermediate range. Similarly, earlier work by Quraishi et al. [6] questioned the contemporary applicability of Tokuhashi scoring systems developed before advances in systemic therapies. More recent studies have demonstrated that traditional scoring systems, such as Tokuhashi and Tomita, show reduced predictive accuracy as oncologic management has evolved with modern systemic therapies [15-16]. Despite these historical limitations, our data suggest that structured scoring systems retain clinical value for identifying patients with poor prognosis, particularly when applied consistently. ChatGPT-assisted predictions based on Tokuhashi criteria achieved comparable performance to spinal surgeons, emphasizing the potential utility of AI models to support-but not replace-clinical decision-making. 

An important limitation is that the different prognostic approaches were not based on identical inputs. Oncologists likely had access to broader longitudinal oncological context, including treatment response, disease trajectory, and remaining systemic therapy options, whereas surgeon-calculated Tokuhashi scores and ChatGPT-based predictions were derived only from the anonymized clinical slides. This may partly explain the better oncologist performance for longer-term survival, while also highlighting the difficulty of prognostication in MSCC.

The slight differences observed between ChatGPT-calculated Tokuhashi scores and surgeon-calculated scores likely reflect differences in rule-based implementation versus clinician-applied judgment. While ChatGPT systematically applied the scoring rules based purely on provided data, surgeons may have incorporated implicit clinical judgment, particularly in ambiguous cases (e.g., borderline functional status or mixed metastatic patterns). However, the small absolute difference in prediction accuracy of ChatGPT-calculated Tokuhashi scores and surgeon-calculated (53% vs. 49%) and similar recall rates for <6 months survival (68% vs. 70%) suggest that these discrepancies were minor and unlikely to be clinically meaningful.

While logistic regression analyses demonstrated strong associations between metastatic cancer burden and survival category, tree-based machine learning models, including random forest, decision tree, and XGBoost, consistently identified Karnofsky Performance Status as the strongest predictor of survival, with age being the second strongest predictor. This difference reflects methodological differences between the statistical measures rather than true inconsistency. Logistic regression coefficients quantify linear log-odds associations per unit change. They can emphasize variables with strong directional effects, such as the presence and count of metastatic cancer. On the other hand, tree-based models capture nonlinear relationships and variable interactions. This led to Karnofsky Performance Status emerging as the dominant variable in the prediction models. We also acknowledge that heterogeneity in primary tumor type may have influenced prediction performance, particularly in the >12-month survival group, where more favorable tumor biology and greater responsiveness to systemic therapy may have contributed to longer survival. Overall, these findings, which should be interpreted cautiously given the small sample size, class imbalance, and lack of external validation, are still consistent with prior observations [6,16]. 

While AI applications in spinal oncology are expanding, most efforts to date have concentrated on imaging classification tasks rather than structured clinical survival prediction [17-18]. In addition, even advanced AI models encounter difficulties when predicting complex survival endpoints, particularly when intermediate time frames are considered [19-20]. Our study addresses this critical gap by applying AI models to real-world structured clinical data in a prognostic context.

Clinically, our findings underscore the persistent difficulty in accurately predicting survival between six and 12 months - a range where surgical decision-making often hinges most critically on life expectancy. The modest performance of all methods in this subgroup reinforces the need for cautious, multidisciplinary evaluation rather than sole reliance on scoring systems or emerging AI tools. At present, AI-assisted prediction can serve as a supportive adjunct to structured human judgment but should not supplant it.

Future research should focus on integrating dynamic, longitudinal clinical data and developing hybrid models that combine oncological, functional, and patient-reported outcomes to improve prognostication. External validation across multiple institutions and prospective testing in clinical decision workflows will be essential to move beyond theoretical performance into meaningful clinical impact. Future studies with larger cohorts may also benefit from tumour-specific or continuous survival modelling to better characterize heterogeneity within the longer-survival group.

Several limitations warrant acknowledgment. First, while data were anonymized and presented consistently, minor ambiguities inherent to real-world clinical documentation may have influenced predictions. Second, although ChatGPT applied literature-based reasoning and Tokuhashi calculation without bias, its predictions are still bounded by the limitations of the data and scoring systems provided. Third, the study cohort, while reflective of typical MSCC referrals, originates from a single tertiary center and may not fully capture broader oncological variability. Fourth, we did not prospectively retain a structured item-level record of individual revised Tokuhashi score components for both surgeon- and ChatGPT-derived assessments. Therefore, although the underlying clinical variables were available on the anonymized slides and the surgeon-calculated Tokuhashi score was removed before presentation to ChatGPT, formal post hoc domain-level comparison between the two approaches could not be performed reliably. In addition, the relatively small sample size may limit the power to detect differences in overall accuracy despite statistical tests being performed. Finally, although a standardized prompt framework was used, large language model outputs may still vary according to prompt phrasing and session context.

## Conclusions

Structured prognostic frameworks, particularly Tokuhashi scoring, continue to provide meaningful guidance in estimating short-term survival among patients with MSCC. Augmentation with AI-assisted calculations, such as those generated by ChatGPT, achieved comparable performance to experienced spinal surgeons in predicting short survival, highlighting the potential role of AI tools as clinical adjuncts. For long-term survival, oncologists performed better. However, intermediate survival prediction remains a critical unmet need across all modalities, reinforcing the necessity for multidisciplinary evaluation and cautious interpretation of prognostic estimates. Functional status and patient age emerged as more reliable survival predictors than traditional oncological variables, suggesting that future prognostic strategies should prioritize dynamic functional metrics over categorical tumor-based scores. Prospective validation and integration of longitudinal clinical data will be essential to refine and personalize survival prediction in spinal oncology.

## Appendices

ChatGPT prompts used for this study

Familiarization/Calibration Prompt

You will be shown anonymized clinical summary slides for patients with metastatic spinal disease.

Before formal predictions begin, this example slide is provided only so that you can familiarize yourself with the structure and formatting of the clinical summaries, including common abbreviations, clinical tables, imaging summaries, and layout conventions.

Please review the slide and confirm that you understand the abbreviations, format, and structure of the information presented.

Do not use this example for analysis in the study dataset. Do not retain or refer back to this case when assessing subsequent patients.

Literature-Based Survival Prediction Prompt

You are reviewing an anonymized clinical summary of a patient with metastatic spinal cord compression.

Based only on the information provided in this slide, estimate the patient’s life expectancy using clinical reasoning and general medical knowledge from the literature. Do not calculate or use a Tokuhashi score unless explicitly asked.

Please consider the following factors where relevant: age, primary tumour type, extent of metastatic disease, neurological status, functional status/Karnofsky Performance Status, imaging findings, prior or current oncological treatment, and other major comorbid or clinical features shown on the slide.

After reviewing the case, assign the patient to one of the following survival categories only: 1) <6 months, 2) six to 12 months, and 3) >12 months.

Please provide 1) the predicted survival category and 2) a brief explanation for the prediction based only on the slide content

Tokuhashi-Based Survival Prediction Prompt

You are reviewing an anonymized clinical summary of a patient with metastatic spinal cord compression.

Based only on the information provided in this slide, independently calculate the revised Tokuhashi score and then assign the predicted survival category according to the Tokuhashi framework.

Please 1) identify the relevant Tokuhashi variables from the slide, 2) calculate the total revised Tokuhashi score, and 3) assign the patient to one of the following survival categories only: <6 months, six to 12 months, and >12 months.

Please provide 1) the individual Tokuhashi components, 2) the total Tokuhashi score, 3) the predicted survival category, and 4) a brief explanation based only on the slide content.
